# Association of Simple Anthropometric Indices and Body Fat with Early Atherosclerosis and Lipid Profiles in Chinese Adults

**DOI:** 10.1371/journal.pone.0104361

**Published:** 2014-08-04

**Authors:** Zhe-qing Zhang, Li-ping He, Xiao-yan Xie, Wen-hua Ling, Juan Deng, Yi-xiang Su, Yu-ming Chen

**Affiliations:** 1 Guangdong Provincial Key Laboratory of Food, School of Public Health, Sun Yat-sen University, Guangzhou, People's Republic of China; 2 Department of Medical Ultrasound, The First Affiliated Hospital, Sun Yat-sen University, Guangzhou, People's Republic of China; 3 Guangzhou Panyu Central Hospital, Guangzhou, People's Republic of China; Temple University School of Medicine, United States of America

## Abstract

**Objective:**

The discriminatory capability of different adiposity indices for atherosclerosis and lipid abnormalities remains uncertain. This study aimed to identify the best adiposity index for predicting early atherosclerosis and abnormal lipid profiles among anthropometric parameters and body fat measures in middle-aged and elderly Chinese.

**Method:**

A total of 2,063 women and 814 men (57.6±5.2 y) were recruited for this community-based cross-sectional study. Body mass index (BMI), waist circumference (WC), hip circumference (HC), waist-to-hip ratio (WHR) and waist-to-height ratio (WHtR) were assessed. Body fat mass and its percentage values for the whole body and trunk were measured by bioelectrical impedance analysis (BIA). The intima-media thicknesses (IMTs) of the common carotid arteries (CCA), internal carotid arteries (ICA) and bifurcation (BIF) were determined via B-mode ultrasound. The fasting lipid profiles were assessed.

**Results:**

With per SD increase of adiposity indices, the magnitude of the changes of IMT values and lipid profiles was more substantial for WC, WHR and WHtR in both genders. A multivariate logistic regression analysis indicated that WC, WHR and WHtR were more sensitive in predicting the presence of intima-media thickening at the three segments as well as the lipids disturbances in women and men. In general, BIA-derived measures have no added predictive value for IMT-thickening as opposed to those three traditional abdominal measures.

**Conclusion:**

Our findings suggest that abdominal anthropometric measures including WC, WHR and WHtR are sensitive for discriminating carotid atherosclerosis and lipids abnormalities. WC is the best index because of its simplicity in routine use.

## Introduction

Obesity has been well documented as a major pathological condition in people predisposed to cardiovascular disease. The standard epidemiologic translation of this important clinical fact has been anthropometrically measured. One meta-analysis showed a 40% increase in vascular-caused mortality for every 5 kg/m^2^ increase in body mass index (BMI) above 25 kg/m^2^ [1]. Another indicated that the relative risk (RR) of a CVD event increased by 2% per 1 cm increase in WC [2]. Despite the clear value of obesity for CVD diagnostic evaluation, researchers have compared the discriminatory capability of different adiposity indicators for the presence of CVD risk, especially between BMI and abdominal obesity measurements [3]–[5]. An appropriate, simple obesity parameter would be valuable in clinical practice for CVD risk estimation.

Obesity is defined as a condition of excess fat mass. However, neither BMI nor body circumference can differentiate fat mass from lean mass. Many sophisticated methods such as dual-energy x-ray absorptiometry (DXA), computed tomography (CT) and magnetic resonance imaging (MRI) have been developed to provide more precise estimates of the location and amount of adipose tissue in various body regions [6]. However, technical difficulties, high costs and a lack of equipment portability limit their use in routine clinical practice and epidemiologic research at large. Bioelectrical impedance analysis (BIA) has been considered a valid alternative for measuring body fat in large studies or clinical practice due to its low cost and easy application, which has also been validated against reference methods [7]. It remains uncertain whether more direct obesity measures have added incrementally useful information to that provided by simple anthropometric assessments taken during cardiovascular risk screenings. A few studies have attempted to address this question in the context of cardiometabolic risks such as hypertension, insulin resistance, dyslipidaemia and metabolic syndrome, but the results are contradictory [8], [9].

Accumulative evidence has proved that subclinical vascular diseases, which could be surrogated by carotid artery wall intima-media thickness (IMT), an early manifestation of atherosclerosis, are strong predictors of future CVD events [10]. Lipid abnormalities play a critical role in the development of atherosclerosis [11]. However, few studies have compared the validity of different simple adiposity indices with measured body fat (or % body fat) in discerning people at particular risk of subclinical atherosclerosis and abnormal lipids profiles. Therefore, this community-based, cross-sectional study aimed to compare the discriminatory performance of simple anthropometric adiposity indices with more direct measurements of obesity derived from BIA to identify Chinese middle-aged women and men at risk of subclinical atherosclerosis and lipid abnormalities.

## Materials and Methods

### Study population

This study was based on the baseline data of a cohort study designed to assess the environmental and genetic determinants of cardiometabolic endpoints and osteoporosis, to which 3,169 participants aged between 50 and 75 years were recruited between July 2008 and June 2010 in Guangzhou, Guangdong Province, China. All participants had been residents of urban Guangzhou for more than 5 years. Apparently healthy participants were recruited by sending invitation letters to residential buildings, by posting local advertisements, by giving health talks and from referrals in the local community. Individuals with confirmed diabetes, cardiovascular disease, renal failure, cancer, metabolic bone disease, chronic glucocorticoid use or a history of spine or hip fracture were excluded. All participants gave written informed consent, and the study was approved by the Ethics Committee of Sun Yat-sen University.

### Data collection

Eligible participants were invited to a local health center or the School of Public Health at Sun Yat-sen University. Trained interviewers conducted face-to-face interviews based on a structured questionnaire to collect socio-demographic characteristics and lifestyle information related to smoking, alcohol intake, and education status. A modified self-administered 79-item FFQ was applied to collect dietary consumption information, as described elsewhere [12]. The dietary total energy was calculated according to the Chinese Food Composition Table, 2002 [13]. Daily physical activity was estimated by a 24-hour physical activity questionnaire containing 19 items [14]. Well-trained research staff performed physical examinations and anthropometric measurements at the time of visit. IMT was measured by sonographers at Sun Yat-sen University's First Affiliated Hospital within 2 months after the first interview.

### Anthropometric measurements

Anthropometric measurements were made while the participants wore light clothing and no shoes. Body weight and height were directly measured according to standard procedures. BMI was calculated as weight in kilograms divided by height in meters squared. WC was measured at the umbilicus level. Hip circumference was measured using the point of maximum girth around the buttocks. Each parameter was measured twice. The waist-to-hip ratio (WHR) was calculated between the two circumferences. The waist-to-height ratio (WHtR) was calculated by dividing waist circumference by height. The absolute and percentages of fat mass in both the whole body and trunk were assessed by a BIA system using a Tanita TBF-418B (Tanita Corporation, Tokyo, Japan). The measurements were performed on each participant in an upright position, with electrodes in contact with the soles and heels of both feet. Biological impedance was measured using four terminals. A 0.8 mA current with a 50 kHz frequency was applied via source electrodes on both soles, and the voltage drop was compared with the heel electrodes. The in-vivo reproducibilities of the BIA system ware 1.54%, 1.85%, 2.50% and 2.79% for the body fat mass (BF),percentage BF (%BF), truck fat mass (TF), and percentage TF (%TF), respectively.

### Carotid intima-media thickness

IMT was measured via B-mode ultrasound with a high-resolution, 7.0–12.0 MHz linear-array transducer system (Aplio TOSHIBA, Japan) in the far walls of three main segments of the right and left extracranial carotid arteries, including the common carotid artery (CCA) (1 cm proximal to dilation of the carotid bulb), bifurcation (BIF) (1 cm proximal to the flow divider) and internal carotid artery (ICA) (1 cm section of the internal carotid artery immediately distal to the flow divider) [15]. The B-mode images at the diastolic phase of the cardiac cycle were recorded by professionals in the ultrasound departments at Sun Yat-sen University's First Affiliated Hospitals, who were blinded from the participants' identity and all other study parameters. The inner and outer walls of the carotid artery were scanned longitudinally to assess the best incidence to obtain a clear image. On a longitudinal, two-dimensional ultrasound image of the carotid artery, the far walls of the artery were displayed as two bright white echogenic lines separated by a hypoechogenic space. The wall thickness was measured manually with computer assistance using an electronic caliper [14]. The average IMT was calculated as the mean of the IMT values from the right and left segments. The thickening of the artery wall was defined as the IMT at either side of at least 1.0 mm [16]. Reproducibility was assessed by replicating measurements for 91 randomly selected participants using a new image on the same day. The intra-reader and inter-reader correlation coefficients were 0.98 and 0.86 for the CCA-IMT, 0.72 and 0.68 for the BIF-IMT and 0.99 and 0.94 for the ICA-IMT, respectively.

### Lipids profiles measurements

The methods for the measurements of fasting plasma lipid profiles including total cholesterol (TC), total triglycerides (TG), low density lipoprotein cholesterol (LDLc) and high density lipoprotein cholesterol (HDLc) were described in a previous report [17]. Lipids disturbances were defined according to the NCEP ATP III criteria: TC ≥6.22 mmol/L, TG ≥2.26 mmol/L, LDLc ≥4.14 mmol/L, or HDLc <1.03 mmol/L [18].

### Statistical analyses

All analyses were carried out using the SPSS statistical package (version 13.0, SPSS Inc., Chicago, IL), and P values below 0.05 were considered significant. All analyses were split according to gender due to the known differences in body composition. Quantitative variables were expressed as mean values ± standard deviation (SD) and categorical data were expressed as frequencies. The obesity measurement variables were separately standardized into normal Z-scores. A multiple linear regression analysis was also applied to determine the change in the average IMT at the CCA, BIF and ICA and lipid profiles per 1-SD increase of WC, WHR and WHtR after controlling for age, education level, smoking and alcohol intake status, energy intake and physical activity. The odds ratios (ORs) and their 95% confidence intervals (CIs) for carotid thickening occurrences along with each additional standard deviation (SD) increase in the examined obesity indices were estimated by logistic regression after adjusting for the aforementioned covariates. Enter procedures were used in all of the regression models.

## Results

### Participant characteristics

A total of 3,169 participants completed the questionnaire survey. Among these, 2,877 participants (2,063 women and 814 men) who completed all of the anthropometric measurement, body fat and IMT determinations were included in the final analysis. The anthropometrics and clinical measurements are summarized in Table 1. The mean age of the participants was 56.8 for women and 59.4 for men. In women, the average (SD) WC and WHR were 80.0(8.9) cm ands 0.87(0.06), respectively, whereas in men they were 86.1(8.4) cm and 0.92(0.05), respectively. On the basis of fat mass, women tended to deposit more fat in the whole body and trunk. The total body fat percentage of women was almost 11% higher than that of men. Among the women participants, 24.7% were overweight and 3.0% were obese according to the BMI criteria. The equivalent estimation in men was 34.9% and 3.3%, respectively. The percentage of participants suffering from artery wall thickening at the CCA, BIF and ICA was 9.6%, 49.0% and 4.9% for women and 21.5%, 67.6% and 11.8% for men, respectively. In terms of lipids disturbances, the percentages of participants have elevated total cholesterol, triglycerides and LDL cholesterol and reduced HDL cholesterol was 24.2%, 14.2%, 28.9, and 9.5% for women and 14.2%, 18.6%, 20.9% and 25.1% for men, respectively.

**Table 1:** Characteristics of the study participants.

	Women		Men	
	Mean±SD, *n*(%)	*N*	Mean±SD, *n*(%)	*N*
Age(year)	56.8±4.9	2063	59.4±5.5	814
Body height (cm)	155.9±5.6	2063	166.5±5.8	814
Body weight (kg)	56.1±8.5	2063	66.0±9.2	814
WC (cm)	80.9±8.8	2063	86.1±8.4	814
HC (cm)	93.2±6.1	2063	93.9±5.3	814
BMI (kg/m^2^)	23.1±3.2	2063	23.8±3.0	814
WHR (m/m)	0.87±0.06	2063	0.92±0.05	814
WHtR (m/m)	0.52±0.06	2063	0.52±0.05	814
BF (kg)	17.9±6.2	1603	13.5±5.0	650
BF% (%)	30.9±6.5	1603	19.9±5.4	650
TF (kg)	9.5±3.9	1603	7.5±3.1	650
TF%(%)	29.5±8.1	1603	20.4±6.7	650
Carotid IMT(mm)				
Mean CCA	0.68±0.14	2063	0.77±0.20	814
Mean BIF	0.89±0.20	2063	1.01±0.30	814
Mean ICA	0.62±0.14	2063	0.69±0.18	814
Lipid profiles(mmol/L)				
TG	1.53±1.02	2004	1.69±1.21	790
TC	5.55±1.06	2004	5.11±1.01	790
LDLc	3.68±0.92	2004	3.47±0.86	790
HDLc	1.44±0.33	2004	1.22±0.28	790
Physical activity (MET⋅h/d)*	31.6±4.0	2063	31.1±4.8	814
Energy intake (kcal/d)	1841±501	2063	2145±573	814
Education, *n*(%)		2063		814
Primary school or below	165(8.0)		47(5.8)	
Secondary school	466(22.6)		187(23.0)	
High school or above	1597(77.4)		580(71.2)	
Smoker, *n*(%)	12(0.6)	2063	413(50.7)	814
Alcohol drinker, *n*(%)	43(2.1)	2063	126(15.5)	814

WC: Waist circumference; HC: hip circumference; BMI: body mass index; WHR: waist to hip ratio; WHtR: Waist to height ratio; BF: body fat mass; TF: trunk fat mass; CCA: common carotid artery; ICA: internal carotidartery; BIF: bifurcation; IMT: intima-media thickness; TC: total cholesterol; TG: triglycerides.

*: Physical activity was evaluated by metabolic equivalent (MET) hours per day.

### Regression coefficients of IMT and lipid profiles with obesity indices

Among the studied obesity indices, WC had the strongest association with IMTs, while WHR was the best predictor of blood lipids in women (Table 2). In men, conventional abdominal obesity measures (WC, WHR and WHtR) generally exhibited stronger associations with IMT values and lipid profiles than BMI and BIA-derived obesity measures (Table 3). Similar results were observed in age-stratified subgroups (Supplemental data: Table S1, Table S2).

**Table 2:** Regression coefficients of intima-media thickness and lipid profiles with obesity indices in women.*

Obesity indices (SD)	Intima-media thickness (mm, ×10^−2^)			Plasma lipids (mmol/L, ×10^−2^)			
	CCA	BIF	ICA	TC	TG	LDLc	HDLc
WC	**2.13±0.30** d	**2.03±0.43** d	**1.60±0.30** d	1.79±2.45	21.2±2.29^d^	7.86±2.11^c^	−11.3±0.70^d^
HC	1.63±0.30^d^	1.25±0.43^b^	1.05±0.30^c^	−2.51±2.39	7.06±2.28^b^	3.62±2.07	−5.83±0.71^d^
BMI	1.91±0.30^d^	1.49±0.43^b^	1.27±0.30^d^	0.12±2.41	16.5±2.28^d^	7.06±2.08^b^	−8.51±0.71^d^
WHR	1.85±0.31^d^	**2.04±0.44** d	1.48±0.31^d^	**5.88±2.50** a	**27.0±2.31** d	**9.17±2.15** d	**−12.2±0.71** d
WHtR	2.00±0.31^d^	1.71±0.44^c^	1.49±0.31^d^	2.72±2.49	21.9±2.33^d^	8.85±2.15^d^	−11.2±0.72^d^
%BF	1.37±0.34^d^	0.78±0.48	0.75±0.34^a^	2.12±2.60	17.6±2.64^d^	7.29±2.29^b^	−8.46±0.77^d^
BF	1.73±0.34^d^	1.02±0.48^a^	1.02±0.34^b^	−0.49±2.60	16.3±2.64^d^	5.53±2.29^a^	−6.75±1.06^d^
%TF	1.21±0.34^c^	0.70±0.48	0.66±0.34	2.46±2.60	16.8±2.64^d^	7.21±2.29^b^	−8.14±0.77^d^
TF	1.52±0.34^d^	0.99±0.48^a^	1.03±0.34^b^	0.58±2.60	15.5±2.65^d^	6.05±2.29^b^	−7.73±0.77^d^

BMI, WC, HC, WHR, WHtR, BF, %BF, TF, %TF, CCA, ICA, BIF, TC, TG: see Table 1.

a : p<0.05;

b : p<0.01;

c : p<0.001;

d : p<0.0001.

* Regression coefficients (beta±SEM).Independent variables: age, education level, smoking and alcohol intake status, energy intake and physical activity, and Z-score of the corresponding obesity index (method entered).

**Table 3:** Regression coefficients of intima-media thickness and lipid profiles with obesity indices in men.*

Obesity indices (SD)	Intima-media thickness (mm, ×10^−2^)			Plasma lipids (mmol/L, ×10^−2^)			
	CCA	BIF	ICA	TC	TG	LDLc	HDLc
WC	**3.79±0.69** d	2.11±0.91^a^	**2.26±0.64** c	12.7±3.62^c^	31.0±4.23^d^	12.0±3.10^c^	−8.60±0.96^d^
HC	3.12±0.69^d^	1.38±0.92	2.07±0.65^b^	11.2±3.65^b^	20.7±4.34^d^	11.2±3.14^c^	−6.62±0.99^d^
BMI	3.43±0.70^d^	**2.43±0.91** b	2.06±0.64^b^	12.3±3.61^b^	30.4±4.22^d^	11.9±3.09^c^	−8.56±0.96^d^
WHR	3.59±0.70^d^	2.18±0.90^a^	1.77±0.64^b^	10.8±3.61^b^	**31.7±4.20** d	9.61±3.09^b^	−8.11±0.96^d^
WHtR	3.68±0.70^d^	2.23±0.91^a^	2.08±0.64^b^	12.6±3.63^b^	30.7±4.25^d^	**12.7±3.10** d	**−9.13±0.95** d
%BF	2.64±0.81^b^	1.68±1.04	1.37±0.74	10.5±3.97^b^	25.3±4.77^d^	8.59±3.43^a^	−8.41±0.76^d^
BF	2.99±0.81^c^	1.78±1.04	1.68±0.74^a^	11.2±3.99^b^	27.0±4.79^d^	8.50±3.45^a^	−6.73±1.06^d^
%TF	2.83±.081^c^	1.46±1.04	0.92±0.74	**13.5±3.96** b	26.8±4.77^d^	10.5±3.42^b^	−6.64±1.06^d^
TF	3.04±0.81^c^	1.54±1.04	1.31±0.74	11.8±3.98^b^	28.0±4.79^d^	8.66±3.45^a^	−6.91±1.06^d^

BMI, WC, HC, WHR, WHtR, BF, %BF, TF, %TF, CCA, ICA, BIF, TC, TG: see Table 1.

a : p<0.05;

b : p<0.01;

c : p<0.001;

d : p<0.0001.

* Regression coefficients (beta±SEM). Independent variables: age, education level, smoking and alcohol intake status, energy intake and physical activity, and Z-score of the corresponding obesity index (method entered).

### Odd ratios of intima-media thickening and lipids disturbances according to obesity indices

In general, all adiposity indices were positively and dose-dependently associated with the risk of carotid intima-media thickening and lipids abnormalities, and the associations were more pronounced in men than in women (Table 4, Table 5). The risk of CCA-IMT thickening was increased by a range of 20% (HC) to 43% (WHR) in women and 40% (%BF) to 71% (WC and WHtR) in men for each SD increase in the various adiposity indices. Similar associations were observed at the ICA-IMT and BIF-IMT, but the associations were less significant than those at the CCA-IMT. In general, the traditional abdominal anthropometric predictor had a more significant association with IMT than any BF, %BF, TF and %TF measured by BIA in both men and women. For the presence of elevated triglycerides and LDL cholesterol, and reduced HDL cholesterol, the ORs were also larger for per 1-SD increase of WC, WHR and WHtR than BMI and the BIA-derived measures regardless of sex. (Table 4, Table 5)

**Table 4:** Odds ratios (95%CI) of intima-media thickening and lipids disturbances according to obesity indices in women.

Obesity indices(SD)	IMT thickening			Lipids disturbances			
	CCA	BIF	ICA	TC	TG	LDLc	HDLc
WC	1.40(1.20–1.63)^c^	1.22(1.14–1.37)^d^	**1.40(1.14–1.73)** b	1.03(0.93–1.15)	1.51(1.32–1.71)^d^	1.25(1.13–1.38)^d^	**1.54(1.32–1.79)** d
HC	1.20(1.04–1.39)^a^	1.10(1.01–1.21)^a^	1.29(1.05–1.57)^a^	0.94(0.84–1.04)	1.13(1.00–1.48)	1.15(1.04–1.26)^b^	1.30(1.13–1.50)^c^
BMI	1.26(1.09–1.46)^a^	1.18(1.08–1.30)^c^	1.33(1.08–1.62)^b^	1.01(0.91–1.13)	1.39(1.23–1.58)^d^	1.25(1.14–1.37)^d^	1.46(1.27–1.69)^d^
WHR	**1.43(1.22–1.67)** d	**1.24(1.13–1.36)** d	1.32(1.06–1.64)^a^	**1.14(1.02–1.27)** a	**1.74(1.52–2.01)** d	1.25(1.12–1.38)^d^	1.52(1.30–1.79)^d^
WHtR	1.36(1.17–1.59)^d^	1.20(1.09–1.32)^c^	1.38(1.12–1.71)^b^	1.07(0.96–1.19)	1.56(1.37–1.78)^d^	**1.28(1.15–1.42)** d	1.53(1.32–1.79)^d^
%BF	1.26(1.06–1.49)^a^	1.09(0.98–1.20)	1.23(0.96–1.57)	1.10(0.98–1.23)	1.43(1.23–1.66)^d^	1.22(1.09–1.79)^b^	1.48(1.21–1.81)^d^
BF	1.27(1.08–1.50)^b^	1.04(1.00–1.22)	1.31(1.04–1.65)^a^	1.02(0.91–1.14)	1.37(1.19–1.58)^d^	1.18(1.06–1.31)^b^	1.43(1.19–1.73)^c^
%TF	1.22(1.02–1.45)^a^	1.08(0.98–1.20)	1.18(0.93–1.51)	1.12(1.00–1.26)	1.42(1.22–1.66)^d^	1.22(1.09–1.37)^c^	1.45(1.18–1.78)^c^
TF	1.26(1.07–1.49)^b^	1.14(1.00–1.23)	1.34(1.06–1.68)^a^	1.05(0.94–1.18)	1.37(1.19–1.57)^d^	1.19(1.06–1.32)^b^	1.37(1.14–1.67)^b^

BMI, WC, HC, WHR, WHtR, BF, %BF, TF, %TF, CCA, ICA, BIF, IMT, TC, TG: see Table 1.

Odds ratios (95%CI): adjusted for age, education level, smoking and alcohol intake status, energy intake and physical activity;

a : p<0.05;

b : p<0.01;

c : p<0.001;

d : p<0.0001.

**Table 5:** Odds ratios (95%CI) of intima-media thickening and lipids disturbances according to obesity indices in men.

Obesity indices (SD)	IMT thickening			Lipids disturbances			
	CCA	BIF	ICA	TC	TG	LDLc	HDLc
WC	**1.71(1.41–2.08)** d	1.17(1.00–1.38)	1.49(1.18–1.89)^b^	1.18(0.96–1.45)	1.63(1.34–1.98)^d^	1.28(1.07–1.53)^b^	**1.69(1.41–2.02)** d
HC	1.50(1.24–1.81)^d^	1.03(0.88–1.22)	1.42(1.13–1.78)^b^	1.18(0.96–1.46)	1.31(1.08–1.58)^b^	1.22(1.02–1.46)^a^	1.55(1.30–1.84)^d^
BMI	1.57(1.31–1.89)^d^	1.10(0.94–1.30)	1.49(1.18–1.86)^b^	1.19(0.97–1.46)	1.63(1.35–1.97)^d^	1.27(1.06–1.51)^b^	1.64(1.39–1.97)^d^
WHR	1.62(1.34–1.97)^d^	**1.25(1.07–1.47)** b	1.36(1.08–1.73)^b^	1.12(0.91–1.38)	**1.76(1.44–1.15)** d	1.25(1.04–1.49)^a^	1.56(1.31–1.87)^d^
WHtR	**1.71(1.41–2.08)** d	1.18(1.00–1.39)	**1.56(1.23–1.99)** c	1.71(0.95–1.44)	1.63(1.34–1.99)^d^	**1.32(1.10–1.59)** d	1.68(1.40–2.02)^d^
%BF	1.40(1.15–1.70)^b^	1.27(1.06–1.53)^a^	1.40(1.09–1.78)^b^	1.09(0.87–1.36)	1.46(1.19–1.79)^c^	1.11(0.92–1.35)	1.40(1.16–1.69)^b^
BF	1.44(1.18–1.74)^c^	1.26(1.05–1.52)^a^	1.44(1.13–1.84)^b^	1.13(0.90–1.41)	1.46(1.20–1.78)^c^	1.14(0.94–1.38)	1.42(1.18–1.71)^d^
%TF	1.47(1.19–1.8)^c^	1.22(1.02–1.46)	1.42(1.10–1.85)^b^	1.11(0.89–1.40)	1.50(1.21–1.86)^c^	1.20(0.98–1.46)	1.43(1.17–1.74)^c^
TF	1.50(1.23–1.83)^b^	1.27(1.04–1.51)^a^	1.48(1.16–1.98)^b^	1.11(0.89–1.39)	1.44(1.18–1.77)^c^	1.17(0.97–1.42)	1.47(1.22–1.78)^d^

BMI, WC, HC, WHR, WHtR, BF, %BF, TF, %TF, CCA, ICA, BIF, IMT, TC, TG: see Table 1.

Odds ratios (95%CI): adjusted for age, education level, smoking and alcohol intake status, energy intake and physical activity.

a : p<0.05;

b : p<0.01;

c : p<0.001;

d : p<0.0001.

## Discussion

This study first compared the discriminative capability of different adiposity indices, including simple anthropometric indices (WC, HC, BMI, WHR and WHtR), BIA-measured body fat and percentage body fat of the whole body and trunk (BF, %BF, TF and %TF), to indicate the presence of carotid intima-media thickening at the CCA, BIF and ICA and the abnormal lipid profiles in Chinese men and women. The results generally showed that the traditional abdominal obesity measures including WC, WHR and WHtR had more robust, highly significant relationships with all of the IMT measurements and lipid profiles in both genders after adjusting for covariates. Those three measures outperformed BIA body fat measurements in predicting artery wall thickening and lipid abnormalities in both genders.

A few cross-sectional studies have compared the discriminatory capability of different adiposity indices in the detection of early atherosclerosis [19]–[26]. However, their findings have been conflicting, with some studies proving BMI to be better than or at least as good as markers of abdominal obesity [20], [22], [25], while others showing that abdominal surrogates afforded better atherosclerotic burden discrimination [23], [26]. Some added the more direct measurement of body fat, but also restricted either the sample size or measured artery wall segments [24]–[26]. Our study importantly extended the analyses of those previous association studies in a relatively larger sample by selecting the most appropriate predictor among indirect and direct adiposity measures for predicting artery wall thickening at different segments. We failed to find the superiority of those more direct measurements of fatness by BIA in predicting early carotid artery wall thickening as well as lipid disturbances. This was consistent with the findings from a sample of 849 Japanese men, in which the intra-abdominal fat area assessed by CT was not superior to BMI or WHR in predicting CCA-IMT [25]. A cross-sectional study of 2924 British men aged 60–79 years showed that BMI and WC were the measures that more strongly associated with the metabolic syndrome than %BFM and BFM (OR per SD, 1.61& 1.65 vs. 1.41 & 1.53) [27]. Another study further demonstrated that the measures of central obesity were more significantly associated with the coronary artery calcium score than either the parameters assessing overall obesity or other more direct measures of visceral adiposity assessed by a computed tomography [28].Our findings and the many above-mentioned other studies suggested that simple anthropometric measurements might do even better than the direct fat measures in the prediction of cardiovascular risk factors.

Although the clinical implications and contributions of abdominal adiposity to global cardiometabolic risk have been reviewed extensively [29], [30], there has been continuous debate over whether WC, WHR or WHtR is a better measure of abdominal adiposity in predicting health risks. The European Prospective Investigation into Cancer and Nutrition in Norfolk cohort suggested that coronary heart disease (CHD) risk estimates for waist circumference without hip circumference adjustment were lower by 10%–8% [31]. However, in the Nurses' Health Study, the BMI- and covariate-adjusted hazard ratios per SD increase in WHR and WC were 1.17 (95%CI: 1.11–1.23) and 1.45 (95%CI: 1.28–1.56), respectively, for CVD mortality [32]. The results from the multi-ethnic case-control INTERHEART study confirmed the stronger association of WHR than BMI with myocardial infarction risk, but WHR has not typically been markedly superior to the use of WC alone [3]. The International Day for the Evaluation of Abdominal Obesity (IDEA) study of 168,000 primary care patients extended the INTERHEART study findings, which involved only myocardial infarction. The study highlighted the prognostic value of WC measurement in terms of CVD [33]. In addition, WHR showed poorer test-retest and intra- and inter-observer reliability than WC measurement alone [34]. Furthermore, successful weight loss interventions commonly reduced the numerator and denominator, resulting in no observed change in the ratio [35]. Comparable WHR values were observed among some extremely lean and obese women and some participants with very low and very high levels of abdominal visceral adipose tissue, which also confirmed the limitations of the ratio measures [36]. WHtR has also recently been proposed as a better predictor in terms of the emergence of cardiometabolic risks such as dyslipidaemia, metabolic syndrome and hypertension, especially among Asians [4], [37], [38]. However, the validity of WHtR was less determinant in terms of cardiovascular event occurrence. Our study generally showed abdominal obesity measures (WC,WHR or WHtR) were superior than BMI and BIA-%BFM and BFM in predicting carotid atherosclerosis and lipids abnormalities. Among these three indices, no one was consistently better than the others for the predictions of IMT thickening and lipid abnormalities. Considering the complicated biologic interpretation the relative complexity of WHR and WHtR, WC might be the best index in routine use.

The IMT measurements at different segments and sites showed differing patterns of association with CVD risk factors. The CCA-IMT was strongly associated with risk factors for stroke, whereas the BIF-IMT was more directly associated with ischemic heart disease risk factors [39]. The ICA-IMT was a stronger independent correlate of incident myocardial infarction and prevalent CVD [40]. Therefore, the screening validity of different adiposity indicators for the presence of artery wall thickening was evaluated in different segments. The magnitude of the correlations was greater between the CCA-IMT and all of the adiposity measures, which was consistent with the previous findings [41].

This study had some limitations. The participants were recruited through advertisements, health talks and subject referrals from community centers and thus did not comprise a random sample, which might attenuate their representativeness. In addition, it should be noted that the direct fat mass indicators were measured using BIA, a method not considered a “gold standard” but rather an appealing alternative to assessing body composition in epidemiological studies. However, BIA has been shown to accurately predict DXA-derived %BF in the Chinese population (*r* = 0.89, *P*<0.05) [42]. The TF% assessed via BIA also showed high correlations with the MRI measurements of the total abdominal adipose tissue (*r* = 0.93 in men and r = 0.89 in women, *P*<0.05) [43].

In conclusion, our findings suggest that WC, WHR, and WHtR is superior than BMI and directly measures of body fat in predicting carotid atherosclerosis and lipids abnormalities, and that BIA-derived fat measures have no added predictive value in middle-aged and elderly Chinese men and women. And WC might be the best index in routine use for screening those risk factors because of its simplicity and sensitivity.

## Supporting Information

Table S1 **Age-stratified regression coefficients of intima-media thickness and lipid profiles with obesity indices in women.**
(DOCX)

Table S2 **Age-stratified regression coefficients of intima-media thickness and lipid profiles with obesity indices in men.**
(DOCX)
