# Supplementary material for: Association of Simple Anthropometric Indices and Body Fat with Early Atherosclerosis and Lipid Profiles in Chinese Adults
Source: PLoS One. 2014 Aug 4;9(8):e104361. doi: 10.1371/journal.pone.0104361 (PMC4121270; doi:10.1371/journal.pone.0104361)
Supplement: Table S1 — Age-stratified regression coefficients of intima-media thickness and lipid profiles with obesity indices in women. (DOCX) [file pone.0104361.s001.docx]

**Table S1**.Age-stratified regression coefficients of intima-media thickness and lipid profiles with obesity indices in women^*^

| Obesity indices (SD) | Intima-media thickness (mm, ×10^-2^) | | | Plasma lipids (mmol/L, ×10^-2^) | | | |
| --- | --- | --- | --- | --- | --- | --- | --- |
|  | CCA | BIF | ICA | TC | TG | LDLc | HDLc |
| **<56 years** |  |  |  |  |  |  |  |
| WC | **2.47±0.39***^d^* | **1.88±0.55***^b^* | 1.08±0.39*^b^* | 3.82±3.41 | 20.8±2.83*^d^* | 9.05±2.90*^b^* | -11.2±1.02*^d^* |
| HC | 2.17±0.39*^d^* | 1.67±0.54*^b^* | 1.26±0.39*^b^* | -0.75±3.37 | 9.03±2.86*^b^* | 4.16±2.88 | -6.44±1.05*^d^* |
| BMI | 2.21±0.39*^d^* | 1.80±0.54*^b^* | 1.13±0.39*^b^* | 3.71±3.38 | 18.2±2.82*^d^* | 8.69±2.88*^b^* | -8.58±1.04*^d^* |
| WHR | 1.76±0.39*^d^* | 1.36**±**0.55*^a^* | 0.45±0.39 | **7.30±3.44***^a^* | **24.0±2.83***^d^* | 10.5±2.93^c^ | **-11.4±1.03***^d^* |
| WHtR | 2.24±0.39*^d^* | 1.70±0.5*^5b^* | 0.83±0.39*^a^* | 5.83±3.43 | 21.8±2.83*^d^* | 9.71±2.92*^b^* | -10.5±1.04*^d^* |
| %BF | 1.55±0.47*^b^* | 1.18±0.64 | 0.57±0.45 | 5.26±3.73 | 18.7±3.30*^d^* | **10.7±3.27***^b^* | -9.18±1.12*^d^* |
| BF | 1.99±0.45*^d^* | 1.38±0.64*^a^* | 1.03±0.45*^a^* | 3.40±3.73 | 18.3±3.30*^d^* | 9.59±3.27*^b^* | -9.33±1.12*^d^* |
| %TF | 1.37±0.46*^b^* | 1.03±0.64 | 0.41±0.45 | 4.65±3.74 | 17.9±3.30*^d^* | 9.36±3.28*^b^* | -9.08±1.12*^d^* |
| TF | 1.67±0.46*^c^* | 1.29±0.64*^a^* | 1.04±0.44*^a^* | 4.69±3.73 | 17.3±3.30*^d^* | 10.7±3.27*^b^* | -8.79±1.12*^d^* |
| **>56 years** |  |  |  |  |  |  |  |
| WC | **2.45±0.46^d^** | 2.52±0.64*^c^* | 2.21±0.43*^d^* | -0.23±3.42 | 20.7±3.49*^d^* | 6.49±3.00*^a^* | -11.2±0.95*^d^* |
| HC | 1.41±0.46*^b^* | 1.01±0.64 | 0.93±0.44*^a^* | -3.48±3.36 | 5.03±3.48 | 3.39±2.95 | -5.31±0.98*^d^* |
| BMI | 1.95±0.47*^d^* | 1.52±0.65*^a^* | 1.49±0.44*^b^* | -2.51±3.40 | 14.6±3.50*^d^* | 6.07±2.99*^a^* | -8.39±0.97*^d^* |
| WHR | 2.43±0.46*^d^* | **3.01±0.64***^d^* | **2.51±0.44***^d^* | 3.53±3.43 | **27.8±3.45***^d^* | 7.07±3.01*^a^* | **-12.3±0.94***^d^* |
| WHtR | 2.35±0.47*^d^* | 2.20±0.65*^b^* | 2.23±0.44*^d^* | -0.13±3.46 | 20.8±3.54*^d^* | **7.70±3.04***^a^* | -11.4±0.96*^d^* |
| %BF | 1.70±0.51*^b^* | 0.87±0.72 | 1.05±0.51*^a^* | 0.05±3.58 | 16.3±4.00*^d^* | 4.67±3.20 | -7.91±1.06*^d^* |
| BF | 1.95±0.51*^d^* | 1.09±0.72 | 1.14±0.51*^a^* | -3.00±3.59 | 14.3±4.03*^c^* | 2.36±3.22 | -7.70±1.16*^d^* |
| %TF | 1.53±0.51*^b^* | 0.79±0.72 | 1.00±0.51 | 1.11±3.57 | 15.3±4.00*^c^* | 5.19±3.20 | -7.43±1.06*^d^* |
| TF | 1.83±0.51*^c^* | 1.09±0.72 | 1.14±0.51*^a^* | -1.96±3.60 | 13.7±4.04*^b^* | 2.45±3.22 | -6.90±1.08*^d^* |

WC: Waist circumference; HC: hip circumference; BMI: body mass index; WHR: waist to hip ratio; WHtR: Waist to height ratio; BF: body fat mass; TF: trunk fat mass; CCA: common carotid artery; ICA: internal carotid artery; BIF: bifurcation; TC: total cholesterol; TG: triglycerides.

*^a^*: p<0.05; *^b^*: p<0.01; *^c^*: p<0.001;*^d^*: p<0.0001.

^*^ Independent variables: age, education level, smoking and alcohol intake status, energy intake and physical activity, and Z-score of the obesity index (method entered)
