# Supplementary material for: Association of Simple Anthropometric Indices and Body Fat with Early Atherosclerosis and Lipid Profiles in Chinese Adults
Source: PLoS One. 2014 Aug 4;9(8):e104361. doi: 10.1371/journal.pone.0104361 (PMC4121270; doi:10.1371/journal.pone.0104361)
Supplement: Table S2 — Age-stratified regression coefficients of intima-media thickness and lipid profiles with obesity indices in men. (DOCX) [file pone.0104361.s002.docx]

**Table S2**. Age-stratified regression coefficients of intima-media thickness and lipid profiles with obesity indices in men^*^

| Obesity indices (SD) | Intima-media thickness (mm, ×10^-2^) | | | Plasma lipids (mmol/L, ×10^-2^) | | | |
| --- | --- | --- | --- | --- | --- | --- | --- |
|  | CCA | BIF | ICA | TC | TG | LDLc | HDLc |
| <59 y |  |  |  |  |  |  |  |
| WC | **3.96±0.84***^d^* | 1.82±1.13 | 2.08±0.77*^b^* | 15.5±5.28*^b^* | 39.5±7.02*^d^* | 8.98±4.56 | -8.63±1.40*^d^* |
| HC | 3.69±0.84*^d^* | 1.32±1.14 | 1.99±0.77*^a^* | 15.7±5.31*^b^* | 24.9±7.23*^b^* | **12.2±4.57***^b^* | -6.98±1.43*^d^* |
| BMI | 3.52±0.84*^d^* | 1.93±1.13 | 1.76±0.77*^a^* | 14.7±5.27*^b^* | 39.9±6.99*^d^* | 9.24±4.55*^a^* | -9.16±1.39*^d^* |
| WHR | 3.16±0.84*^c^* | 1.81±1.12 | 1.59±0.76*^a^* | 11.2±5.25*^a^* | **42.1±6.89***^d^* | 4.13±4.53 | -8.10±1.30*^d^* |
| WHtR | 3.35±0.84*^d^* | 1.58±1.13 | 1.49±0.77 | 14.1±5.27*^b^* | 40.1±6.98*^d^* | 9.10±4.54*^a^* | **-9.25±1.37***^d^* |
| %BF | 2.95±0.97*^b^* | 2.95±1.26*^a^* | 1.79±0.85*^a^* | 12.1±6.05*^a^* | 35.5±8.40*^d^* | 5.56±5.23 | -7.11±1.62*^d^* |
| BF | 3.50±0.97*^c^* | 2.84±1.28*^a^* | 2.07±0.86*^a^* | 17.6±6.05*^b^* | 38.7±8.43*^d^* | 7.47±5.27 | -6.92±1.64*^d^* |
| %TF | 3.12±0.97*^b^* | **3.15±1.26***^a^* | 1.82±0.84*^a^* | 18.1±5.98*^b^* | 37.2±8.36*^d^* | 10.9±5.19*^a^* | -7.45±1.61*^d^* |
| TF | 3.47±0.97*^c^* | 3.13±1.27*^a^* | **2.21±0.86***^a^* | **19.4±6.03***^b^* | 39.1±8.42*^d^* | 9.95±5.25 | -7.47±1.63*^d^* |
| >59 y |  |  |  |  |  |  |  |
| WC | **3.89±1.12***^b^* | 2.38±1.43 | 2.25±1.02*^a^* | 8.96±5.02 | **22.7±5.03***^d^* | 13.1±4.26*^b^* | -8.60±1.32*^d^* |
| HC | 2.69±1.13*^a^* | 1.46±1.44 | 1.98±1.03 | 5.77±5.06 | 16.7±5.12*^b^* | 9.45±2.31*^a^* | -6.36±1.36*^d^* |
| BMI | 3.16±1.13*^b^* | 2.90±1.43*^a^* | 2.14±1.03*^a^* | 9.01±5.03 | 21.9±5.05*^d^* | 12.6±4.28*^b^* | -8.12±1.33*^d^* |
| WHR | 3.80±1.12*^b^* | 2.47±1.42 | 1.78±1.02 | 9.88±5.00*^a^* | 21.6±5.02*^d^* | 13.3±4.25*^b^* | -8.01±1.33*^d^* |
| WHtR | 3.80±1.13*^b^* | 2.83±1.44 | **2.42±1.03***^a^* | **9.96±5.05***^a^* | 21.6±5.08*^d^* | **14.1±4.28***^b^* | **-9.01±1.33***^d^* |
| %BF | 2.22±1.27 | 0.56±1.62 | 0.86±1.17 | 7.99±5.31 | 16.3±5.23*^b^* | 10.6±4.60*^a^* | -6.88±1.39*^d^* |
| BF | 2.32±1.28 | 0.90±1.63 | 1.15±1.18 | 4.68±5.36 | 16.7±5.26*^b^* | 8.54±4.64 | -6.97±1.40*^d^* |
| %TF | 2.44±1.27 | 0.11±1.63 | 0.08±1.18 | 9.07±5.31 | 18.3±5.22*^b^* | 9.86±4.61*^a^* | -6.37±1.40*^d^* |
| TF | 2.47±1.27 | 0.26±1.63 | 0.43±1.18 | 4.55±5.35 | 17.9±5.24*^b^* | 7.08±4.64 | -6.91±1.39*^d^* |

BMI, WC, HC, WHR, WHtR, BF, %BF, TF, %TF, CCA, ICA, BIF, TC, TG: see Table S1.

*^a^*: p<0.05; *^b^*: p<0.01; *^c^*: p<0.001;*^d^*: p<0.0001;

^*^ Independent variables: age, education level, smoking and alcohol intake status, energy intake and physical activity, and Z-score of the obesity index (method entered)
